# *In vitro* Antimicrobial Activity of Acne Drugs Against Skin-Associated Bacteria

**DOI:** 10.1038/s41598-019-50746-4

**Published:** 2019-10-10

**Authors:** Mark A. T. Blaskovich, Alysha G. Elliott, Angela M. Kavanagh, Soumya Ramu, Matthew A. Cooper

**Affiliations:** Institute for Molecular Bioscience, The University of Queensland, Brisbane, Queensland 4072 Australia

**Keywords:** Acne vulgaris, Antimicrobial resistance, Antibiotics

## Abstract

Acne is a common skin affliction that involves excess sebum production and modified lipid composition, duct blockage, colonization by bacteria, and inflammation. Acne drugs target one or more of these steps, with antibiotics commonly used to treat the microbial infection for moderate to severe cases. Whilst a number of other acne therapies are purported to possess antimicrobial activity, this has been poorly documented in many cases. We conducted a comparative analysis of the activity of common topical acne drugs against the principal etiological agent associated with acne: the aerotolerant anaerobic Gram-positive organism *Propionibacterium acnes* (recently renamed as *Cutibacterium acnes*). We also assessed their impact on other bacteria that could also be affected by topical treatments, including both antibiotic-sensitive and antibiotic-resistant strains, using broth microdilution assay conditions. Drugs designated specifically as antibiotics had the greatest potency, but lost activity against resistant strains. The non-antibiotic acne agents did possess widespread antimicrobial activity, including against resistant strains, but at substantially higher concentrations. Hence, the antimicrobial activity of non-antibiotic acne agents may provide protection against a background of increased drug-resistant bacteria.

## Introduction

Acne vulgaris is a common skin disease^1^ that affects almost all teenagers and many adults to a degree. It is estimated as the eighth most prevalent global disease, with 650 million people reported to have had acne in 2010^2^. The development of acne proceeds in four stages, starting with excess sebum production and modified lipid composition in the sebaceous gland at the base of hair follicles, which is followed by blocking of the skin pore, then colonization by *Propionibacterium acnes* (recently renamed as *Cutibacterium acnes*, but with the original designation still favored in the dermatological community^3^), which induces inflammation and pustule formation^1,4,5^. Treatment options include skin cleansing to remove excess oil and unblock pores, skin abrasives (including chemical peeling agents such as benzoyl peroxide **1**, azelaic acid **2**, salicyclic acid **3** or retinoids) to increase cell turnover and help remove lesions, hormones or retinoid treatment to reduce sebum production, and antibiotics to reduce the bacterial infection^1,6^.

**Figure 1:**
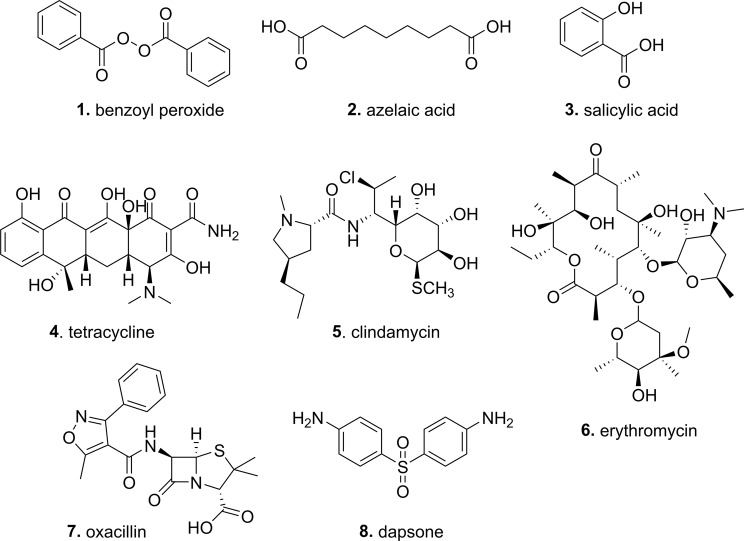
Structures of antibiotics and anti-acne agents tested.

Antibiotics prescribed for acne can be topical or systemic. For systemic treatment, usually reserved for more severe acne, the oral tetracyclines (tetracycline **4**, oxytetracycline, doxycycline, minocycline or lymecycline) are most commonly used^1^. Oral clindamycin **5** is effective but has adverse effects, while macrolides (erythromycin **6** and azithromycin), trimethoprim and the β-lactams ampicillin/amoxicillin/oxacillin **7** are discouraged due to concerns over growing resistance^1,7^. The use of systemic antibiotics, other than the tetracyclines and macrolides, is not recommended due to limited data supporting their use to treat acne^7^. Topical antibiotic options include tetracycline **4**, clindamycin **5**, and erythromycin **6**, (see Fig. 1), sometimes in combination with benzoyl peroxide **1** and zinc acetate^1^. Dapsone (diaminodiphenyl sulfone) **8** (see Fig. 1), an anti-inflammatory agent with antimicrobial properties, has also been used. However, a number of other topical agents are proposed to act via multiple mechanisms, with the exfoliants benzoyl peroxide **1**, azelaic acid **2**, and salicylic acid **3** (see Fig. 1) commonly ascribed to also have antimicrobial activity. Surprisingly, there is little literature evidence of the extent of their antimicrobial activity, particularly under standardized broth microdilution assay conditions.

The growing global crisis of antibiotic resistance is also reflected in antimicrobial acne therapy. Resistant strains of *P*. *acnes* have been reported in many countries (with resistance especially noted to topical erythromycin and clindamycin), and topical antibiotic use is associated with resistance in other commensal bacteria, such as *Staphylococcus aureus*^8^. The American Academy of Dermatology recommends that systemic antibiotic use should be limited to the shortest possible duration with re-evaluation at 3–4 months to minimize the development of bacterial resistance, and co-application of benzoyl peroxide (BP) to help reduce the development of resistance^7^. Topical therapy is strongly suggested to follow the discontinuation of systemic antibiotics as a maintenance regimen^7^. The European Evidence-Based Guideline for the Treatment of Acne has similar recommendations^9^.

The rise in antimicrobial resistance is accompanied by an increasing awareness of the role of the human microbiome in the ability of pathogenic species to establish an infection. Several recent genomic studies have specifically examined the human skin microbiome^10–17^, and even the subpopulation of *P*. *acnes* in the human skin microbiome^18,19^. These studies identify abundant populations of *P*. *acnes* and *Staphylococcus* spp. (especially *S*. *epidermidis*, but also *S*. *aureus* and *S*. *hominis*, and lower levels of *S*. *warneri*, *S*. *saprophyticus*, *S*. *lugdunensis*, *S*. *haemolyticus* and *S*. *capitis*). High levels of Corynebacterium, *Streptococcus mitis* and the fungus *Malassezia globosa* have also been identified, with community composition varying depending on the skin region and skin type (sebaceous, dry skin or wet skin)^8,13^. The top ten bacteria present in sebaceous sites are *P*. *acnes*, *S*. *epidermidis*, *Corynebacterium tuberculostearicum*, *S*. *capitis*, *Corynebacterium simulans*, *S*. *mitis*, *S*. *hominis*, *Corynebacterium aurimucosum*, *Corynebacterium kroppenstedtii*, and *Corynebacterium amycolatum*^13^. Lactobacillus, β-Proteobacteria and Flavobacteriales are also found in sebaceous sites, though usually are more common in ‘dry’ or ‘moist’ skin environments^10^. Altering the skin microbiome with topical antibiotic treatment can have significant effects on the cutaneous host defense^20^, and some skin bacteria (such as *Micrococcus luteus*) have been found to enhance *S*. *aureus* pathogenesis^21^. A new anti-acne tetracycline, sarecycline, has been designed as the first narrow-spectrum tetracycline-class antibiotic being developed for acne treatment, reducing collateral damage on the microbiome (though in this case used systemically, not topically)^22^.

It is important to know the relative effects of antimicrobial agents on human microbiota in order to understand their potential to foster resistance and alter the microbiome composition. To date, there has not been a comparative assessment of the antimicrobial activity of commonly used antibiotics and topical acne agents against a set of representative commensal skin bacteria, including those not directly associated with acne. We now report such a study against standardized accessible organisms from reference collections, testing both specific antibiotics used to treat acne (tetracycline, erythromycin, clindamycin, oxacillin, dapsone, along with control antibiotics vancomycin/colistin) and other acne agents reported to have antimicrobial activity (salicyclic acid, azelaic acid, benzoyl peroxide) (see Fig. 1). These are assessed against both sensitive and resistant bacterial strains, under anaerobic and aerobic conditions. In addition to some of the most common strains identified by microbiome studies, we also include several additional pathogenic bacteria that can be found on the skin and/or involved in skin infections, such as Streptococci (*S*. *pyogenes* and, less commonly *S*. *pneumoniae*^23^), Bacilli (*B*. *subtilis*, *B*. *cereus* and *B*. *megaterium*), Enterococci (*E*. *faecium* and *E*. *faecalis*), Micrococci (*M*. *luteus* and *Kocuria rosea*) and the Gram-negative bacteria *Escherichia coli* and *Acinetobacter johnsonii*^24^.

## Results and Discussion

The antimicrobial activity of the antibiotics and anti-acne agents, tested under standard broth microdilution (BMD) conditions, are summarized in Tables 1–3. The topical acne therapeutics originally developed as specific antimicrobial agents (tetracycline, erythromicin, oxacillin, and clindamycin) generally showed potent activity under both anaerobic and aerobic conditions against a range of bacteria, though erythromycin, oxacillin and clindamycin lost substantial activity against resistant bacteria, such as MRSA (methicillin-resistant *S. aureus*) and MDR (multidrug-resistant) *S*. *pneumoniae*. Dapsone, an aniline sulfone first made in 1908 but discovered as an antimicrobial agent in 1937^25^, was generally less effective than the other antibiotics but had widely varying activity that was dependant on the species (ranging from <2 µg/mL for *S*. *pyogenes* to >4100 µg/mL against a *S*. *epidermidis* strain, with the variable activity potentially partly due to poor solubility when diluting from stock solutions into media at high concentrations). Previous literature reports for broth Minimum Inhibitory Concentration (MIC) potency of tetracycline, erythromycin and clindamycin against *P*. *acnes* also showed a wide variation against 16 strains, with activity ranges of ≤0.06 to 31, ≤0.25 to >1000, and ≤0.125 to >500 µg/mL respectively for the three antibiotics^26^, with results from the current study generally fitting into these ranges.

**Table 1:** Minimum Inhibitory Concentrations measured under anaerobic conditions, µg/mL^*^.

Anti-acne agent Bacteria	*antibiotics*						*non-antibiotics*		
	Vancomycin	Tetracycline	Erythromycin	Oxacillin	Clindamycin	Dapsone	Salicylic acid	Azelaic acid	Benzoyl peroxide 75%
*P*. *acnes* ATCC 6919	0.25–1	0.125–1	0.25	0.25–1	0.125	4100	4000–8000	4000–8000	1024–>2048
*A*. *acidipropionici* ATCC 25562	0.125	0.5	0.25–4	0.25–1	0.03–0.125	1025–>4100	500–8000	4000–16000	1024–>2048
*C*. *granulosum* ATCC 25564	0.25	0.25	0.125–2	0.25–4	0.03–0.25	512–>4100	2000–8000	4000–8000	1024–>2048
*S*. *aureus*, MRSA ATCC 43300	1	0.25	>32	8–64	>32	>4100	4000–8000	2000–8000	2048–>2048

[n = 4, duplicate results from 2 independent assays.]

**Table 2:** Minimum Inhibitory Concentrations against Gram-Positive bacteria measured under aerobic conditions, µg/mL.

Anti-acne agent Bacteria	*antibiotics*						*non-antibiotics*		
	Vancomycin	Tetracycline	Erythromycin	Oxacillin	Clindamycin	Dapsone	Salicylic acid	Azelaic acid	Benzoyl peroxide 75%
**Staphylococci**									
*S. aureus*, MSSA ATCC 25923	1	0.25–0.5	1	0.125–0.25	0.125	256	32000	16000	>2048–2048
*S. aureus*, MSSA ATCC 29213	1	0.5	1	0.25–0.125	0.06–0.03	512–1024	64000	16000	2048
***S. aureus***, **MRSA** **ATCC 43300**	1	0.25	>32	16	>32	128–256	32000	16000	2048
***S. aureus***, **MRSA** **ATCC 33591**	1	>32	>32	>64	>32	>4100	64000	16000	≥2048
***S. aureus***, **GISA** **NRS1**	4	32	>32	>64	>32	256–512	32000	8000	2048
***S. aureus***, **VRSA** **VRS1**	>64	1	>32	>64	>32	512–025	32000	2000–16000	2048
*S. capitis* ATCC 27840	1–2	32	0.5	0.06–0.125	0.06–0.125	128	4000	8000–6000	2048
*S. epidermidis* ATCC 12228	1	≥32	0.5	0.125	0.06	>4100	8000	8000	2048
*S. epidermidis* ATCC 14990	1/2	16–32	0.25–0.5	0.03–0.06	0.03	128	2000–4000	16000	2048
***S. epidermidis***, **VISE** **NRS60**	4	32	>32	8	≤0.015	256–1025	8000–16000	8000–16000	2048
*S. warneri* ATCC 27836	1	0.5	0.5	0.5	0.03	4	32000	16000	2048
**Other organisms**									
*B. cereus* ATCC 11778	1	≤0.015	0.25	>64	0.5	256	32000	8000–16000	2048
*B. megaterium* ATCC 13632	0.125	0.5	0.25	0.25–0.5	32	64	16000–32000	8000–16000	2048
*B. subtilis* ATCC 6633	0.06–0.125	0.06–0.125	0.125	0.25	1	4–8	32000	8000–16000	2048
*E. faecium* ATCC 35667	0.5–1	0.5–0.25	2–4	16	≤0.015	>4100	32000	16000	≥2048
*E. faecalis* ATCC 29212	2	32	2–4	8	16	16	32000	16000	1024–2048
*K. rosea* ATCC 31251	1–2	32–16	0.25	0.06–0.12	0.03–0.06	128–256	2000	2000–16000	2048
*M. luteus* ATCC 4698	0.06–0.25	0.06–0.125	0.25	2–4	0.015–0.125	256	2000–4000	4000	1024
*S. pneumoniae* ATCC 33400	1	0.125–0.25	0.015–0.5	0.25	0.06	256–512	32000	8000	2048
***S. pneumoniae***, **MDR** **ATCC 700677**	1	>32	>32	>64	>32	>4100	32000	16000	2048
*S. pyogenes* ATCC 14289	0.25–0.125	0.06	≤0.015	≤0.03	≤0.015	≤2	1000–2000	4000	2048

^*^[n = 4, duplicate results from 2 independent assays; MIC variations indicated. Bacterial species in bold are resistant.]

**Table 3:** Minimum Inhibitory Concentrations against Gram-Negative bacteria measured under aerobic conditions, µg/mL.

Anti-acne agent Bacteria	*antibiotics*						*non-antibiotics*		
	Colistin	Tetracycline	Erythromycin	Oxacillin	Clindamycin	Dapsone	Salicylic acid	Azelaic acid	Benzoyl peroxide 75%
*E*. *coli* ATCC 25922	–	1–2	>32	2	1	>4100	16000	16000	2048
*A*. *johnsonii* ATCC 17909	64	>32–16	0.5	0.06	0.015–0.03	256	2000	8000–16000	2048

[n = 4, duplicate results from 2 independent assays.]

In sharp contrast, the ‘non-antibiotic’ acne agents (salicylic acid, azelaic acid and benzoyl peroxide) that are believed to help treat acne by multiple mechanisms, including bacterial inhibition, had substantially lower, but measurable, activity, compared to true antibiotics. Their potency, generally ranging from 2000-64,000 µg/mL, was approximately 1000-fold less active than the designated antibiotics. However, their activity was maintained against all of the resistant bacteria tested, including highly resistant strains of *S*. *aureus*, *S*. *epidermidis*, and *S*. *pneumoniae* where almost all the antibiotics failed.

Previous reports of the direct antimicrobial activity of salicyclic acid are limited, with disc diffusion measurements of activity in 1962 versus *E*. *coli*, *Aerobacter aerogenes*, *Leuconostoc mesenteroides* P-60, *S*. *aureus*, ‘*Streptococcus faecalis*’ [sic] and five fungi^27^. In 2014 the MIC and Minimum Bactericidal Concentration (MBC) of salicylic acid and other phytochemicals were assessed against *E*. *coli* (MIC = 3200 µg/mL) and *S*. *aureus* (MIC = 1600 µg/mL)^28^, compared to MIC = 16000 µg/mL and 32000–64000 µg/mL, respectively in this study. A 2007 study showed 5 mM salicylate (approx. 700 µg/mL) halted growth of SH1000 *S*. *aureus* after 5h^29^, though the same concentration only slightly slowed the growth of *E*. *coli* GC4468^30^. A 2011 article on new antimicrobial formulations compared their activity against *P*. *acne* to salicyclic acid, with MIC_90_ for salicyclic acid of 1000 µg/mL^26^, compared to 8000 µg/mL in this study. A review of the effects of salicylate on bacteria was published in 2000^31^, which summarized research showing that, at concentrations that do not substantially affect bacterial growth, salicylate can: (a) induce antibiotic resistance, (b) reduce resistance to some antibiotics; and (c) affect production of bacterial virulence factors. More recent studies have supported the reduction in susceptibility of organisms such as *S*. *aureus*^29^ or *Salmonella enterica* serovar Typhimurium^32^ to common antibiotics or antiseptics in the presence of salicylate. Further studies are warranted to see if topical use of salicylate for acne reduces the effectiveness of topical acne antibiotics.

The antimicrobial potential of azelaic acid has been more thoroughly studied than that of salicyclic acid, with a review in 1993^33^. The first observation that it exerted a bacteriostatic effect on aerobic and anaerobic bacteria (including Propionibacterium) appeared as a comment in a 1983 clinical report^34^. A clinical trial noted a 224-fold reduction in the population of Micrococcaceae and 30-fold decrease in the density of *Propionibacterium sp*. on the skin after application of 20% azelaic acid cream (compared to no effect from tetracycline)^35^. Another report measured MIC in broth at pH 6.0 against *S*. *epidermidis*, *S*. *capitis*, and *S*. *hominis* (125 mM, approximately 23,500 µg/mL, similar to our values of 8000-16,000 µg/mL), *P*. *acne* and *P*. *granulosum* (>250 mM ≈ >47,000 µg/mL, versus 8000–16,000 µg/mL in this study), *Propionibacterium avidum* (31 mM ≈ 5900 µg/mL) and *Pityrosporum ovale* (now known as *Malassezia ovale*) (250 mM ≈ 47,000 µg/mL)^36^. In 1991 concentrations of 500 mM (≈ 94,000 µg/mL) were reported to exert bactericidal activity against *P*. *acne in vitro* at pH 6.0, with activity enhanced by lowering the pH to 5.6 but little activity at pH 7.0^37^. A 1992 report compared the *in vitro* activities of the topical antimicrobials azelaic acid, nitrofurazone, silver sulphadiazine and mupirocin against MRSA^38^. Against 80 MRSA strains, the MIC_50_ and MIC_90_ of azelaic acid, measured by agar dilution, were 850 µg/mL and 1150 µg/mL respectively (no pH mentioned), with a range of 600–1200 µg/mL^38^, around 10-fold less than our BMD MIC values (8000–16,000 µg/mL). The corresponding MBC values were 1800 µg/mL and 3500 µg/mL respectively. Azelaic acid was slowly bactericidal at 2500 µg/mL, with around 3-log reduction from a starting inoculum of 10^6^ cfu after 24 h; a resistance mutation rate of <1 × 10^−9^ was observed^38^. The authors of the 1993 review also noted in the review that they had conducted an *in vitro* experiment to assess the development of resistance in *P*. *acnes* or *S*. *epidermidis* over 53 days exposed to 2–4 mM (400–800 µg/mL) azelaic acid, with no changes in MIC detected^33^.

Finally, benzoyl peroxide has long been known to have antimicrobial properties, with speculation of antiseptic action in the 1920’s and treatment of acne/skin lesions in the 1930’s^39^. The history of its application for the treatment of acne was reviewed in 1987^40^ and 2009^39^. Survival curves *of S*. *epidermidis*, *S*. *capitis*, *S*. *hominis*, *P*. *acne*, *P*. *granulosum*, *P*. *avidum* and *P*. *ovale* have been measured in the presence of 10^−2^ – 10^−4^ w/v% benzoyl peroxide, with bacteria showing varying sensitivity but all killed at the higher concentrations^41^. Another study looked at 10 sensitive and 10 erythromycin resistant strains of *P*. *acne*, *P*. *granulosum*, *P avidum*, and 10 sensitive and 10 erythromycin resistant strains of *S*. *epidermidis*, with benzoyl peroxide agar dilution MIC of 64–128 µg/mL and 512 µg/mL respectively^42^ (compared to BMD MIC of 2048 µg/mL in this study; their benzoyl peroxide parent solution had 5% w/w benzoyl peroxide but also contained carbomer 940, 14% alcohol, sodium hydroxide, dioctylsodium sulphosuccinate and fragrance). In 1989 MICs against nine *P*. *acne* strains were reported to be between 100–800 µg/mL^43^ using a modified broth with added 2%Tween and glycerol to improve benzoyl peroxide solubility, a 2 × 10^4^ innoculum, and four day incubation (compared to 2048 µg/mL in this study with BHI broth, 5 × 10^5^ innoculum, and 48 h incubation). It was also not clear what form of benzoyl peroxide was used in the 1989 report, as it was “obtained from commercial products” so likely contained other components. More potent BMD MICs of 62.5, 15.6 and >100 µg/mL were reported against *P*. *acne*, *S*. *aureus* and *S*. *epidermidis* in 2009, again employing different assay conditions from our study that included varied incubation times (72, 24 and 48 h respectively)^44^. A comparison of the activity of new antimicrobial formulations against *P*. *acne* used benzoyl peroxide as a standard, with MIC_90_ for benzoyl peroxide of 50 µg/mL^26^ (compared to 2048 µg/mL determined in this study). A 2016 study assessed the activity of benzoyl peroxide against 44 clinical isolates of *P*. *acne* using the Decker modified broth, with MIC_50_ = 128 µg/mL and MIC_90_ = 256 µg/mL. MBCs were similar to MICs, and a time kill assay showed 5-log reduction in cfu after 1 h at two-fold MIC^45^.

In summary, this study clearly demonstrates that acne agents used primarily for their skin exfoliating properties do indeed have modest, but widespread, antimicrobial activity against a range of skin-associated bacteria, at least when tested in broth microdilution assays. Many skin-related bacteria can form biofilms, which are notoriously more resistant to antimicrobial therapies than vegetative bacteria. The exfoliant topical agents are generally applied at concentrations up to 20-fold higher than topical antibiotics (though in some cases at equivalent concentrations), so they are likely to exert substantial antimicrobial effects despite their reduced antimicrobial potency. Benzoyl peroxide is used as 2.5–10% solutions in gel, cream, lotions or liquid^46^, azelaic acid as 15–20% lotions^46^, and salicylic acid in a range of concentrations (with 0.5–2% commonly used, but up to 10% employed for acne treatments: 2% is the maximum strength allowed in over-the-counter acne products in the United States). Clindamycin, erythromycin and tetracycline topical treatments are generally in the 1–4% range^43–47^, with dapsone used in a 5% gel^46^. The retention of high levels of antimicrobial activity by salicylic acid, azelaic acid and benzoyl peroxide against antibiotic-resistant strains of bacteria suggests that these treatments could be useful alternatives to antibiotic-based therapies in the case of resistant bacteria, and should be further explored as preferred alternatives to prescribed antibiotics to help reduce the development of resistance.

## Methods

### Compound preparation

Stock solutions of compounds were prepared in different solvents at different concentrations, depending on solubility and expected activity range, as presented in Table 4.

**Table 4:** Compounds assayed.

Compound Name	Supplier/Batch	MW	Solvent	Stock Solution Concentration (mg/mL)	Concentration range tested (μg/mL)
Azelaic acid	Alfa Aesar Cat# 36308 Batch 5002P21N	188.22	100% DMSO	640	32,000–15
Benzoyl peroxide 75%	Sigma Cat# 517909–5 G Batch mkbr5398v	242.22	100% DMSO	40.97	2,048–1
Clindamycin hydrochloride	Sigma Cat# PHR1159-1G Batch P500159	424.98	H_2_O	3.21	32–0.015
Colistin sulfate	Sigma Cat# C4461 Batch 018K1151	1155.4	H_2_O	1.28	64–0.03
Dapsone	Sigma Cat# 46158-250 mg Batch SZBC072XV	248.3	100% DMSO	82	4,100–2*
Erythromycin	Sigma Cat# E5389-5G Batch 011M1510V	733.93	20% DMSO	3.20	32–0.015
Oxacillin sodium salt hydrate	Sigma Cat# O1002-1G Batch 018K0610	401.43	H_2_O	3.20	64–0.03
Salicylic acid	Sigma Cat# A5376-100G	138.12	100% DMSO	640	32,000–15
Tetracycline hydrochloride	Sigma Cat#T7660-5G Batch PDS-064-048	480.90	H_2_O	3.20	32–0.015
Vancomycin	Sigma Cat# 861987 Batch 087K0694	1485.71	H_2_O	1.28	64–0.03

^*^poor solubility at >512 μg/mL.

### Minimum Inhibitory Concentration (MIC) determinations

Bacterial strains were purchased from the American Type Culture Collection (ATCC) or Network on Antimicrobial Resistance in *Staphylococcus aureus* (NARSA) (see Table 5).

**Table 5:** Bacterial strains assayed.

Species	Strain	Strain designation
*Acinetobacter johnsonii*	ATCC 17909	Bouvet and Grimont NCTC10308 Type strain, isolated from duodenum
*Bacillus cereus*	ATCC 11778	Frankland and Frankland FDA strain PCI 213
*Bacillus megaterium*	ATCC 13632	De Bary KM
*Bacillus subtilis*	ATCC 6633	subsp. *spizizenii* Nakamura *et al*. NRS 231
*Enterococcus faecium*	ATCC 35667	(Orla-Jensen) Schleifer and Kilpper-Balz LRA 55 03 77 quality control strain
*Enterococcus faecalis*	ATCC 29212	(Andrewes and Horder) Schleifer and Kilpper-Balz isolated from urine
*Escherichia coli*	ATCC 25922	(Migula) Castellani and Chalmers FDA strain Seattle 1946
*Micrococcus luteus*	ATCC 4698	(Schroeter) Cohn Type strain
*Kocuria rosea* (*formerly Micrococcus roseus*)	ATCC 31251	(Flugge) Stackebrandt *et al*. M-1054-1
*Cutibacterium acnes (formerly Propionibacterium acnes)*	ATCC 6919	Scholz and Kilian NCTC 737 Type strain, isolated from facial acne
*Acidipropionibacterium acidiproprionici (formerly Propionibacterium acidipropionici)*	ATCC 25562	VPI 0399 [14 × ] Type strain
*Cutibacterium granulosum (formerly Propionibacterium granulosum)*	ATCC 25564	Scholz and Kilian VPI 0507 Type strain
*Staphylococcus aureus*	ATCC 25923	subsp. aureus Rosenbach Seattle 1945, MSSA
*Staphylococcus aureus*	ATCC 29213	subsp. aureus Rosenbach Wichita, MSSA, isolated from wound
*Staphylococcus aureus*	ATCC 43300	subsp. aureus Rosenbach F-182, MRSA
*Staphylococcus aureus*	ATCC 33591	subsp. aureus Rosenbach 328, MRSA
*Staphylococcus aureus*	NRS1 (ATCC 700699)	subsp. aureus Rosenbach Mu50, VISA/MRSA
*Staphylococcus aureus*	VRS1 (NR-46410)	VRSA
*Staphylococcus capitis*	ATCC 27840	subsp. capitis Kloos and Schleifer, LK 499 Type strain
*Staphylococcus epidermidis*	ATCC 14990	(Winslow and Winslow) Evans Fussel [NCTC 11047] Type strain
*Staphylococcus epidermidis*	ATCC 12228	(Winslow and Winslow) Evans FDA strain PCI 1200
*Staphylococcus epidermidis*	NRS60 (NR-45891)	VISE
*Staphylococcus warneri*	ATCC 27836	Kloos and Schleifer AW 25 Type strain, isolated from human skin
*Streptococcus pneumoniae*	ATCC 33400	(Klein) Chester NCTC 7465 Type strain
*Streptococcus pneumoniae*	ATCC 700677	(Klein) Chester Slovakia 14-10 MDR Resistant to erythromycin, penicillin, and tetracycline, Sensitive to rifampin rifampicin and rifamycin AMP
*Streptococcus pyogenes*	ATCC 14289	Rosenbach C203 S clinical isolate

### Standard aerobic MIC Assay

The compounds along with standard antibiotics were serially diluted with Mueller Hinton broth (MHB) (Bacto laboratories, Cat. No 211443) two-fold across the wells of 96-well standard Polystyrene plates (Corning 3370). For antibiotics not initially dissolved in water, the highest solvent (DMSO) concentration in the final assay solution was 2%. Solvent controls have shown that this concentration does not interfere with bacteria growth. All bacteria strains were cultured in MHB at 37 °C overnight. A sample of each culture was then diluted 40-fold in fresh MHB and incubated at 37 °C for a further 2–3 h. The resultant mid-log phase cultures were diluted in MHB and added to each well of the compound-containing 96-well plates to give a final cell density of 5 × 10^5^ CFU/mL. All the plates were covered and incubated at 37 °C for 24 h. MICs were determined as the lowest concentration showing no visible growth by eye. Assays were conducted in duplicate, with two independent assays (n = 4).

### Standard anaerobic MIC Assay

The MIC assay for anaerobic growth conditions was performed to the same procedure as the standard aerobic MIC assay described above with the following exceptions:

All steps were performed in a COY type B anaerobic chamber with the anaerobic atmosphere controlled by the introduction of 10%CO_2_/5% H_2_ in N_2_CoA gas mix, catalyst Stak-Pak and O_2_-H_2_ gas analyzer, with H_2_ levels kept at ~2% for the duration of the assay. Brain Heart Infusion (BHI) (OXOID CM1135B) media with 1% cysteine to further promote an anaerobic environment was used in replacement of MHB, and this broth was incubated in the anaerobic chamber for 24 h prior to use to allow sufficient atmosphere exchange. All the plates were covered and incubated at 37 °C for 48 h. MICs were determined as the lowest concentration showing no visible growth by eye.
